# Sijunzi decoction alleviates inflammation and intestinal epithelial barrier damage and modulates the gut microbiota in ulcerative colitis mice

**DOI:** 10.3389/fphar.2024.1360972

**Published:** 2024-04-08

**Authors:** Hailun Li, Xing Pu, Yongtao Lin, Xinxin Yu, Jing Li, Lin Bo, Hongwu Wang, Yong Xu, Xiang Li, Donghui Zheng

**Affiliations:** ^1^ Department of Nephrology, Affiliated Huai’an Hospital of Xuzhou Medical University, Huai’an, Jiangsu, China; ^2^ School of Nursing and Midwifery, Jiangsu College of Nursing, Huai’an, Jiangsu, China

**Keywords:** Sijunzi decoction, gut microbiota homeostasis, inflammation, ulcerative colitis, intestinal epithelial barrier

## Abstract

**Ethnopharmacological relevance:**

As a representative classical prescription, Sijunzi decoction has powerful therapeutic effects on spleen–stomach qi insufficiency. Ulcerative colitis (UC) is a chronic, diffuse, and non-specifically inflammatory disorder, the etiology of which still remains unclear. In the traditional Chinese medicine (TCM) perspective, splenic asthenia is the primary cause of UC. Based on this, Sijunzi decoction has been extensively used in TCM clinical practice to alleviate UC in recent years. However, the pharmacological mechanism of Sijunzi decoction in modern medicine is still not completely clear, which limits its clinical application.

**Aim of the study:**

The purpose of this study was to investigate the Sijunzi decoction’s curative effect on acute UC mice and probe into its potential pharmacological mechanism.

**Materials and methods:**

The UC mouse model was set up by freely ingesting a 3% dextran sulfate sodium (DSS) solution. The relieving role of Sijunzi decoction on UC in mice was analyzed by evaluating the changes in clinical parameters, colon morphology, histopathology, inflammatory factor content, intestinal epithelial barrier protein expression level, and gut microbiota balance state. Finally, multivariate statistical analysis was conducted to elucidate the relationship between inflammatory factors, intestinal epithelial barrier proteins, and gut microbiota.

**Results:**

First, the research findings revealed that Sijunzi decoction could visibly ease the clinical manifestation of UC, lower the DAI score, and attenuate colonic damage. Moreover, Sijunzi decoction could also significantly inhibit IL-6, IL-1β, and TNF-α while increasing occludin and ZO-1 expression levels. Subsequently, further studies showed that Sijunzi decoction could remodel gut microbiota homeostasis. Sijunzi decoction was beneficial in regulating the levels of Alistipes, Akkermansia, Lachnospiraceae_NK4A136_group, and other bacteria. Finally, multivariate statistical analysis demonstrated that key gut microbes were closely associated with inflammatory factors and intestinal epithelial barrier proteins.

**Conclusion:**

Sijunzi decoction can significantly prevent and treat UC. Its mechanism is strongly associated with the improvement of inflammation and intestinal epithelial barrier damage by regulating the gut microbiota.

## Graphical Abstract

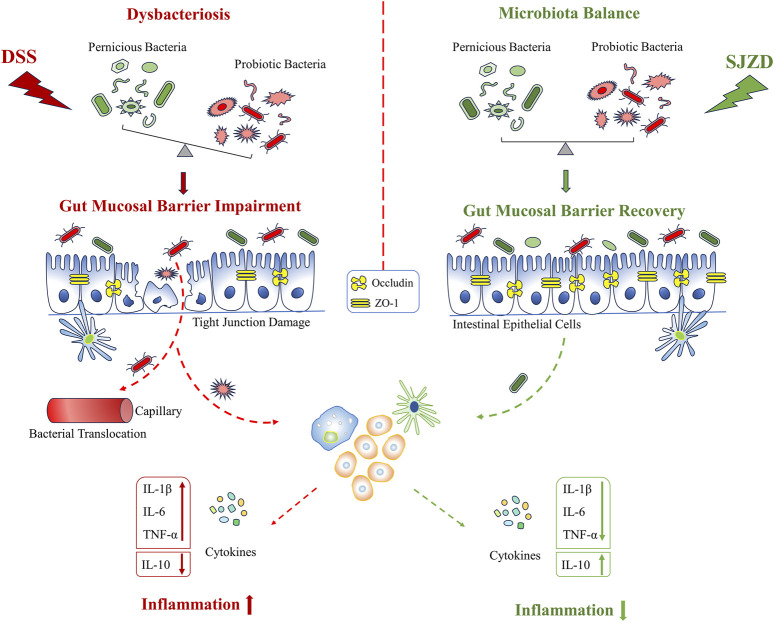

## 1 Introduction

Ulcerative colitis (UC) is a major type of inflammatory bowel disease (IBD), and it is a chronic, diffuse, and unspecific inflammatory state of the human gastrointestinal tract with unknown etiology (Chams et al., 2019). Clinically, UC usually manifests as intermittent diarrhea, abdominal discomfort, tenesmus, bloody or purulent feces, and body weight loss (Tian et al., 2021). Multiple etiologies are involved in UC pathogenesis and progression, such as environmental and luminal factors, hereditary predisposition, and mucosal immune disorders (Kobayashi et al., 2020). So far, a variety of therapeutic drugs, for example, immunomodulators, aminosalicylic acid, glucocorticoids, biologics, and even surgeries, have been used to treat UC (Hirten and Sands, 2021). However, these treatment strategies are often accompanied by adverse reactions, drug resistance, and unsatisfactory clinical curative efficacy (Hirten and Sands, 2021; Liu et al., 2022). Hence, it is imperative to find a novel, safe, and more effective treatment approach.

Due to its safety and effectiveness, traditional Chinese medicine (TCM) has been receiving increasing attention in the clinical practice of gastrointestinal diseases. There is no ‘UC’ in ancient Chinese medicine books. According to the clinical manifestations, there are many ways to name the disease, such as diarrhea, intestinal wind, dysentery, and dirty poison (Liu et al., 2022). From the perspective of TCM, spleen-qi insufficiency is the principal pathogenesis of UC (Tian et al., 2021). Sijunzi decoction from the Song Dynasty’s ‘Tai Ping He Ji Ju Fang’ is a celebrated classic formula in TCM for strengthening the spleen, containing four kinds of Chinese botanical drugs, namely, *Panax ginseng* C. A. Mey. (Renshen in Chinese), *Atractylodes macrocephala* Koidz. (Baizhu in Chinese), *Wolfiporia cocos* (F.A. Wolf) Ryvarden & Gilb. (Fuling in Chinese), and *Glycyrrhiza uralensis* Fisch. ex DC. (Gancao in Chinese) (Wang et al., 2020). Hence, Sijunzi decoction, as a basic prescription to invigorate the spleen and qi, is a preferred choice for the therapy of UC (Lu et al., 2017; Tian et al., 2021). Sijunzi decoction has recently been extensively and widely utilized in the clinical practice of TCM and has been proven to be beneficial for UC (Wu et al., 2023). Nevertheless, the potential mechanism of Sijunzi decoction in preventing UC has not been elucidated.

The gut microbiota plays a pivotal role in disease development, especially in multifarious gastrointestinal disorders (Guo et al., 2020; Feng et al., 2022; Wang et al., 2023). The composition of the gut microbiota in UC patients changes dramatically (Franzosa et al., 2019; Zhang et al., 2020; Zhang et al., 2021). An increase in pathogenic microorganisms combined with a decrease in probiotics has been identified as one of the characteristic features of UC (Dai et al., 2021). Gut microbiota disorder, the imbalance between pathogenic bacteria and normal flora, will damage the epithelial barrier. Then, the weakened barrier would cause bacterial translocation, which further aggravates epithelial barrier disturbance (Zhang et al., 2020; Guo et al., 2023). These processes would form a vicious cycle, resulting in further deterioration of UC. At present, the regulation of the gut microbiota has become a crucial step in UC therapeutic options. Studies have shown that Sijunzi decoction and its main chemical metabolites, including polysaccharides and saponins, can not only regulate the gut microbiota but also affect intestinal bacterial metabolites (Gao et al., 2018; Shi et al., 2019). It is inferred that Sijunzi decoction may have an anti-UC effect by remodeling the gut microbiota. However, there is still a need to experimentally assess this hypothesis.

The effectiveness of Sijunzi decoction in the prevention and treatment of UC was thoroughly assessed in this study. Furthermore, its potential mechanism was also systematically demonstrated from the perspective of remodeling gut microbiota homeostasis in improving epithelial barrier function and reducing inflammation. Both theoretical and clinical studies were carried out in this study to fully illustrate the above information.

## 2 Materials and methods

### 2.1 Materials and reagents

The Chinese medicinal materials used in Sijunzi decoction were sourced from the Bozhou Medicinal Materials Market in Anhui Province, China, and met the standards set by the Pharmacopoeia of the People’s Republic of China. Interleukin-10 (IL-10), tumor necrosis factor-α (TNF-α), interleukin-6 (IL-6), and interleukin-1β (IL-1β) were purchased from Meimian Biotechnology (Yancheng, Jiangsu Province, China). Dextran sulfate sodium (DSS, molecular weight: 36–50 kDa) was obtained from Santa Ana MP Biomedicals in California, USA. Occludin and ZO-1 monoclonal antibodies were bought from Santa Cruz Biotechnology companies in California, USA. The water and methanol used in the experiment were of HPLC grade. Ultrapure water was obtained by filtration of distilled water using a Milli-Q system (Millipore, USA). LC–MS-grade acetonitrile was purchased from Thermo Fisher Scientific (Fair Lawn, New Jersey, USA), and LC–MS-grade formic acid was purchased from Sigma-Aldrich (St. Louis, Missouri, USA). RNAex Reagent, Evo M-MLV RT Mix Kit, and SYBR@ Green Premix Pro Taq HS qPCR Kit were acquired from Accurate Biotechnology (Changsha, China).

### 2.2 Preparation of the Sijunzi decoction extract

**FIGURE 1:**
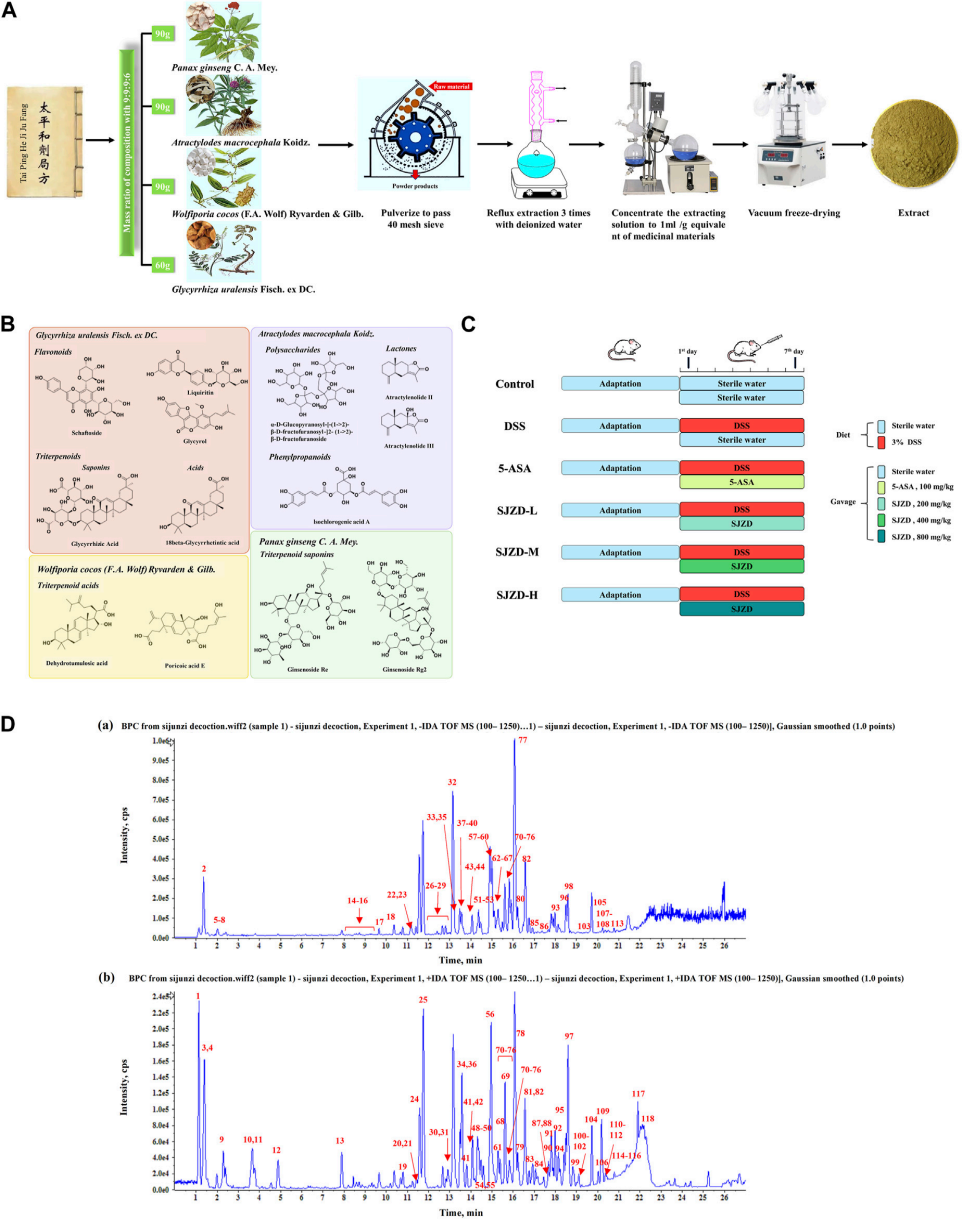
Preparation procedure of SJZD and experimental design. **(A)** Standardized preparation procedure for SJZD. **(B)** Characteristic chemical structure formula. **(C)** Schematic illustration of the experimental design for SJZD administration. **(D)** Base peak chromatogram of SJZD: **(a)** negative ions and **(b)** positive ions.

The four kinds of Chinese botanical drugs, including *Panax ginseng* C. A. Mey. (90 g), *Atractylodes macrocephala* Koidz. (90 g), *Wolfiporia cocos* (F.A. Wolf) Ryvarden & Gilb. (90 g), and *Glycyrrhiza uralensis* Fisch. ex DC. (60 g), following a 9:9:9:6 ratio were pulverized to pass a 40-mesh sieve and then extracted by reflux three times with deionized water (material: solvent = 1:10, m:v, g/mL). Next, the extraction solution was concentrated to 1 mL/g equivalent of medicinal materials. Finally, the concentrated extract was vacuum freeze-dried to obtain the Sijunzi decoction (SJZD) extract. To sum up, a standardized manufacturing process was established by our research group (Figure 1A).

### 2.3 Instruments and conditions

To ensure the quality of the SJZD extract, the UHPLC-QTOF/MS method was used in our research group. Chromatographic separation was performed on an ExionLC system (AB Sciex, Foster City, CA, USA). A Waters ACQUITY UHPLC HSS T3 Column (2.1 × 100 mm, 1.8 μm) was applied at a temperature of 35°C. The mobile phase A was water with 0.1% formic acid (v/v), and B was acetonitrile. The gradient was optimized as follows: 0–5 min from 3% to 8% B, 5–11 min from 8% to 30% B, 11–20 min from 30% to 80% B, 20–21 min from 80% to 95% B, 21–27 min at 95% B, then back to the initial ratio of 3% B, and maintained for an additional 10 min for re-equilibration. The injection volume of all samples was 2 μL.

To provide high-resolution detection, an X500B QTOF Mass Spectrometer (AB Sciex, Foster City, CA, USA) equipped with an electrospray ionization source (turbo ion spray) was applied. MS detection was implemented in both negative and positive ion modes. The parameters of the mass spectrometer were summarized as follows: gas1 and gas2, 45 psi; curtain gas, 35 psi; heat block temperature, 550°C; ion spray voltage, −4.5 kV in the negative mode and 5.5 kV in the positive mode; declustering potential, 60 V; collision energy, ±35 V; and the collision energy spread (CES) was ±15 V.

### 2.4 Animals

Male BALB/c mice (18–22 g) at 6 weeks of age were obtained from the Xuzhou Medical University Animal Center. Animals were fed in transparent plastic cages on a 12-h diurnal light cycle, and simultaneously, the surrounding environment was controlled with humidity (50%–55%) and temperature (22°C ± 2°C). All mice received sterile water and a standard diet during the first week (the adaptation period). Animal experimental procedures followed the rules established by the Experimental Animal Ethics Committee of Xuzhou Medical University (Ethics Approval Number: L20210226229).

### 2.5 UC model and intervention

After acclimatization feeding for 1 week, all mice were randomly distributed into six groups (n = 9), namely, the control group, model group (DSS group), positive group (5-ASA group), and low- (SJZD-L), medium- (SJZD-M), and high- (SJZD-H) dose groups of SJZD (Figure 1C).

Before the experiment, the administration dosages of SJZD and 5-ASA were first determined. Based on the yield of SJZD and its clinical dosage, the dosage in mice was determined according to the human-to-mouse dose conversion principle. Details are as follows: the daily clinical dosage of SJZD for adults is 33 g, and the dose per unit weight for a 70-kg human is approximately 47 mg/kg. Referring to the pharmacological experimental methodology edited by Professor Shuyun Xu, the equivalent dose coefficient between mice and humans was 9.1. Thus, the dose of SJZD administered to mice is calculated as follows: A = 47 (mg kg^−1^) × 9.1 × Z (where Z is the yield of SJZD). The calculations were rounded approximately to give a final result of 400 mg/kg. Then, the exact dose of SJZD is B = A ×W (where W is the body weight of the mice: 18–22 g). Moreover, SJZD of 400 mg/kg was considered the medium-dose group in this study. According to the two-fold correlation, half of the medium-dose group comprised the low-dose group, while the high-dose group was twice the size of the medium-dose group. As a result, the doses administered to SJZD-L, SJZD-M, and SJZD-H mice were 200, 400, and 800 mg/kg of SJZD, respectively. Meanwhile, the dose of 5-ASA was set to 100 mg/kg based on the previous literature (Le Berre et al., 2020).

During the experiment, to set up the mouse model of UC, the control group took standard food and sterile water, while the other five groups took standard food and sterile water containing 3% DSS (w/v, g/mL) (Wirtz et al., 2017). At the same time, SJZD-L, SJZD-M, and SJZD-H groups were administered SJZD of 200, 400, and 800 mg/kg/d by gavage (10 mL/kg/d), respectively. The positive group (5-ASA group) was given 100 mg/kg/d 5-ASA. The control and model (DSS group) groups were fed the same amount of sterile water. The intervention period lasted for 7 days. During this experiment, body weight, bloody stools, and loose stools were noted daily. All mice were anesthetized by inhalation of an overdose of isoflurane on the eighth day. After removing the eyeballs to collect blood, the mice were sacrificed using cervical dislocation. Immediately afterward, the colon tissues of mice were promptly dissected. The colonic length was measured and photographed for recording. Then, one part of the colon was fastened to a 4% concentration of paraformaldehyde, and the other was immediately reserved in an −80°C refrigerator for the next research.

Furthermore, the colonic permeability of UC mice was evaluated by oral administration of fluorescein isothiocyanate (FITC)-labeled dextran (FD-4, MW, 4 kDa) at 10 mg/kg. The mice were deprived of liquid and fasted for 4 h on the eighth day and were subsequently given intragastric administration with a 10 mg/kg FITC-dextran solution. Serum samples were collected after 4 h to measure fluorescence strength using a microplate reader (SpectraMax i3x) (Dong et al., 2022a).

### 2.6 Disease activity index

The same method previously outlined in the literature was used to determine the disease activity index (DAI) score (Hua et al., 2021). According to the recorded results of body weight, bloody stools, and loose stools, the DAI values were scored separately against each rating scale. DAI scores are described as follows: (a) body weight loss scoring criteria: 0 = no body weight loss; 1 = lose weight S ≤ 5%; 2 = loss of weight 5 < S ≤ 10%; 3 = lose weight 10 < S ≤ 15%; and 4 = over 15% weight loss. (b) bloody stools scoring criteria: 0 = no abnormalities; 2 = hemoccult test positive; and 4 = gross bloody stool. (c) stool consistency scoring criteria: 0 = no abnormalities; 1 = fluffy but shaped; 2 = loose stool; 3 = quite floppy and shapeless; and 4 = liquid stools or diarrhea.

The DAI score was evaluated based on formula (1):

$$\text{DAI}=\frac{S_{1}+S_{2}+S_{3}}{3}.$$ (1)

In formula (1), S_1_ indicates the body weight loss score, S_2_ represents the diarrhea score, and S_3_ shows the bloody stool score.

### 2.7 Histological evaluation

Colon tissues were fixed to paraformaldehyde, dewatered, embedded in paraffin wax, and sectioned with a microtome. The obtained slices were dyed with hematoxylin and eosin (H&E), encapsulated airtight with resinene, then observed under a light microscope and blindly assessed by a gastrointestinal expert. Similarly, the scoring for colonic damage was carried out using a previously reported method (Erben et al., 2014).

### 2.8 Cytokine detection

After homogenizing the tissues with precooled PBS, the supernatant fluid was gathered after centrifugation at 3,000 × g for 15 min at 4 °C. The cytokines, labeled as IL-6, TNF-α, IL-10, and IL-1β, were measured using an appropriate ELISA Kit (Meifan Biotechnology, China), following the manufacturer’s product instruction manuals.

### 2.9 Immunofluorescence assay

Colon tissue sections were dewaxed with xylene and successively hydrated with various alcoholic concentrations (100%, 95%, 85%, and 75%) for 10 min. The obtained slices were repaired with citrate antigen, followed by blocking with BSA (0.1%) and Triton X-100 for half an hour. Then, they were rinsed three times with PBS and cultivated at 4 °C overnight with occludin/ZO-1 antibody diluted 1: 200. Tissues were further cultivated with the Alexa Fluor^®^488 antibody, which lasted 2 hours at ambient temperature in the dark. At last, these slices were dyed with 4,6-diamino-2-phenylindole for 5 minutes and then examined using a microscope.

### 2.10 Real-time PCR for mRNA expressions

Total RNA from tissues and cells was extracted using TRIzol. Reverse transcription was conducted using an RT-PCR kit, and then the RT-qPCR was performed on the CFX96 Real-Time PCR Detection System (Bio-Rad, USA). The expressions of target genes were measured using the 2^−ΔΔCT^ method, and the expressions of β-actin were used as an internal control. The mouse primer sequences were designed as follows: β-actin: GGC​TGT​ATT​CCC​CTC​CAT​CG (forward) and CCA​GTT​GGT​AAC​AAT​GCC​ATG​T (reverse); occludin: TGA​AAG​TCC​ACC​TCC​TTA​CAG​A (forward) and TGA​AAG​TCC​ACC​TCC​TTA​CAG​A (reverse); and tight junction protein 1 (TJP1): GCC​GCT​AAG​AGC​ACA​GCA​A (forward) and GCC​CTC​CTT​TTA​ACA​CAT​CAG​A (reverse).

### 2.11 Gut microbiota analysis

Analysis of colonic contents deposited at −80 °C was obtained with 16S rRNA sequencing from Gidio Biotech Co., Ltd. (Guangzhou, China). In brief, DNA in stool samples was extracted using DNA extraction kits and determined on a nanodrop spectrophotometer. 16S rRNA (V3–V4 region) was amplified by polymerase chain reaction with the primers (341F: CCTACGGGNGGCWGCAG; 806R: GGACTACHVGGGTATCTAAT). Amplification products were purified and detected with a platform named NovaSeq from San Diego Illumina Inc. Sequences with 97% similarity were OTU-clustered using UPARSE software. Species composition and relative abundance were analyzed using HemI software (v1.0.3.7). The OTU abundance table (Supplementary Table S1) was used to draw a Venn diagram to analyze the similarity and specificity of OTU composition. Species abundance (ACE, Chao l index) and diversity (Shannon, Simpson index) of the gut microbiota among samples were described by an α-diversity analysis. The reliability of the number of samples for sequencing data was assessed using dilution curves. Non-metric multidimensional scaling (NMDS) analysis was used to compare the otherness in the gut microbial structure. The influence of differential microorganisms between groups was determined by linear discriminant analysis (LEfSe analysis) (LDA score (Log(10) = 2)). Enteric microbiota function was evaluated using Kruskal–Wallis analysis and Wilcoxon analysis.

### 2.12 Statistical analysis

The experiment data were analyzed using GraphPad Prism 9.5.1 and SPSS 17.0. and expressed in mean ± standard deviation form. The intergroup difference was estimated using a one-way ANOVA, followed by Tukey’s multiple comparison test. A multivariate statistical analysis was applied to test the correlation, with statistical significance denoted by *p* < 0.05.

## 3 Results

### 3.1 Basic characterization of the chemical metabolites of SJZD

In order to ensure the quality of SJZD, the UHPLC-QTOF/MS method was used to analyze and identify the complex metabolites of botanical drugs. Unknown metabolites were classified and assigned according to the cleavage rules and diagnostic ions of plant metabolites of different structural types. A total of 118 metabolites were identified in SJZD. A total of 67 chemical metabolites were detected in *Glycyrrhiza uralensis* Fisch. ex DC., mainly flavonoids such as liquiritin and triterpenoids such as glycyrrhizic acid. Seventeen metabolites were detected in *Panax ginseng* C. A. Mey., all of which were triterpene saponins such as ginsenosides. A total of 18 metabolites were detected in *Atractylodes macrocephala* Koidz., including lactones (such as atractylenolides), polysaccharides, and other chemical types. Eight chemical metabolites were extracted from *Wolfiporia cocos* (F.A. Wolf) Ryvarden & Gilb., including triterpene acids such as tumulosic acid. In addition, some common chemical metabolites, such as amino acids and nucleosides, were detected. The base peak chromatogram (BPC) of SJZD is shown in Figure 1D. The numbers of metabolites are marked in the figure, and the corresponding chemical composition characterization results are shown in Supplementary Table S2. The representative chemical-type structural formula is shown in Figure 1B.

### 3.2 SJZD relieved clinical symptoms

**FIGURE 2:**
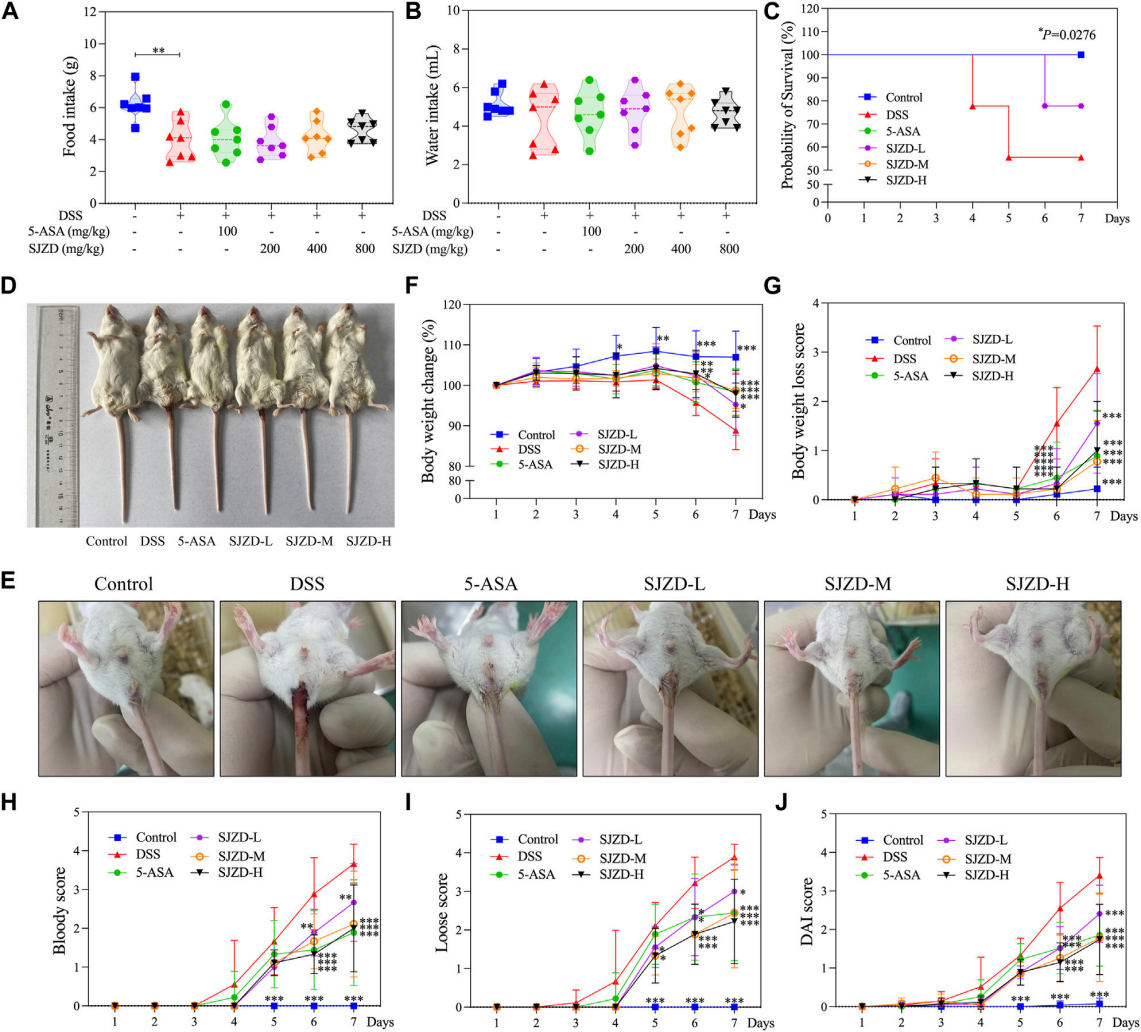
Relieving effect of SJZD on the symptoms. **(A)** Food ingestion **(G)**. **(B)** Water ingestion (mL). **(C)** Probability of survival (%). **(D)** Representative images of animals. **(E)** Picture of a loose stool remaining in the anus. **(F)** Body weight change (%). **(G)** Body weight loss score. **(H)** Bloody score. **(I)** Loose score. **(J)** DAI score. The data were denoted as the mean ± SD (n = 9). *p < 0.05, **p < 0.01, and ***p < 0.001 in comparison with the DSS group.

The UC mouse model was set to drink a 3% DSS solution freely for 7 days (Figure 1C). Food intake was significantly reduced in the DSS group relative to the control group, and there was no obvious difference (*p* > 0.05) in water intake (Figures 2A, B). A survival study revealed that DSS had a much lower survival rate than other groups (Figure 2C). In Figures 2D,F, G, mice in the DSS group displayed a conspicuous decrease in weight compared to those in the control group; however, it could be significantly reversed by 5-ASA or SJZD treatment. The mice in the 5-ASA or SJZD (SJZD-L, SJZD-M, and SJZD-H) group reduced their loss on days 6 and 7 relative to the DSS group, especially in the 5-ASA, SJZD-M, and SJZD-H groups. The DAI score comprehensively reflected weight loss, hematochezia, and diarrhea scores. Therefore, DAI is a significant characteristic indicator for determining the severity of UC. In Figures 2E, H-J, the mice in the DSS group had significant increases in the diarrhea score and bloody stool score compared to those in the control group. As a result, the DAI score for the DSS group was manifestly higher than that of the control group, and simultaneously, the pathological indications evoked by DSS could be effectively reversed by 5-ASA or SJZD interventions. In addition, the therapeutic outcome of SJZD showcased an apparent dose-dependent relationship. The above results implied that the clinical symptoms of UC could be effectively reduced by SJZD.

### 3.3 SJZD alleviated colonic injury

**FIGURE 3:**
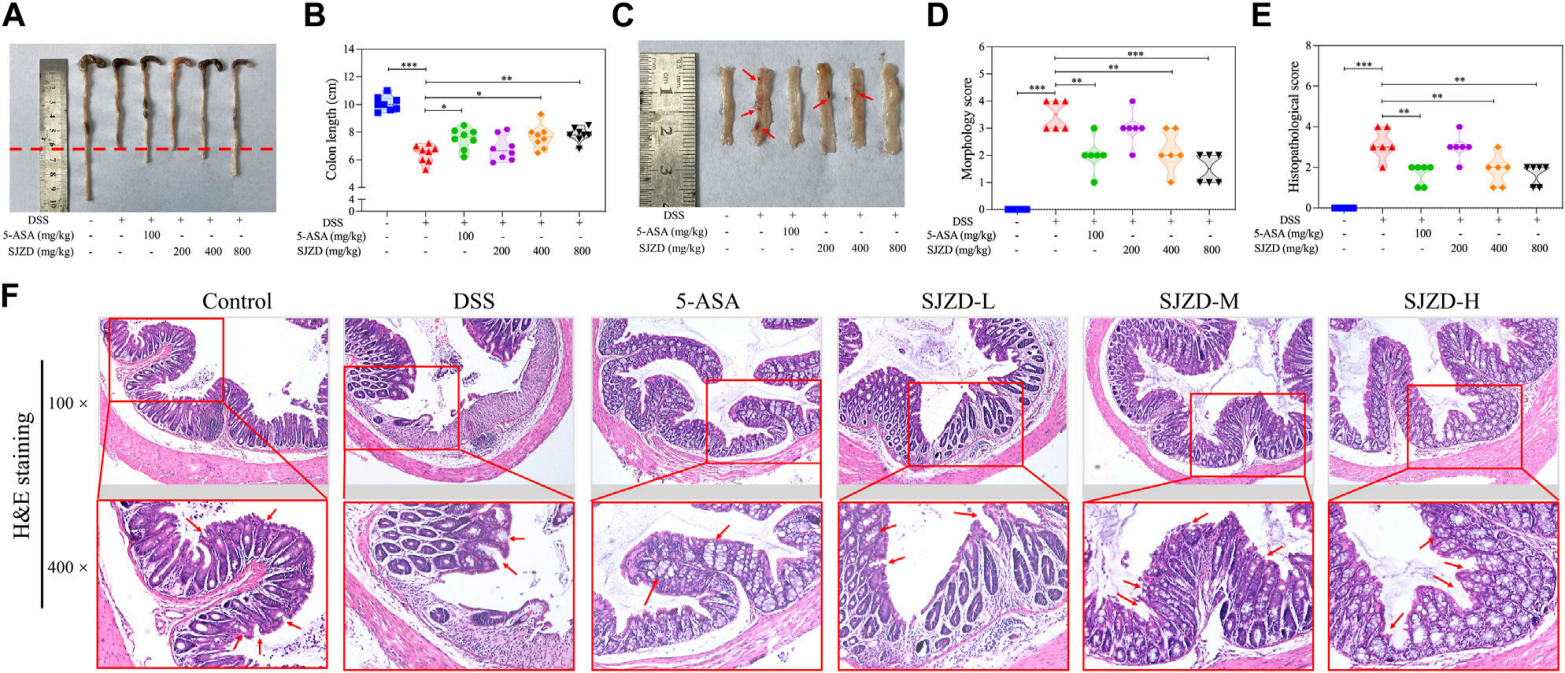
Ameliorating effect of SJZD on colonic damage. **(A)** Representational colonic length pictures. **(B)** Statistical pictures of the colon length. **(C)** Typical colonic injury images. **(D)** Morphology score. **(E)** Histopathological score. **(F)** Colon tissue H&E staining. *p < 0.05, **p < 0.01, and ***p < 0.001 relative to the DSS group. Red arrows indicate edema, adhesions, and ulceration in Supplementary Figure S3C. Red arrows indicate goblet cells and the morphology of crypts in Supplementary Figure S3F.

DSS intervention can cause colon injury and atrophy, which are common phenomena in DSS-induced UC models (Hua et al., 2021). From Figures 3A, B, by comparison of the controls, the colonic length was markedly shortened in the DSS group, while 5-ASA or SJZD treatment inhibited the colonic atrophy, especially in the SJZD-H group, which showed the best inhibitory effect. From Figures 3C, D, it was observed that the DSS group exhibited more severe colon injuries compared with the controls, including edema, adhesions, and ulceration, whereas the severity of these colon injuries induced by DSS was reversed by 5-ASA or SJZD treatment. Meanwhile, histopathological analysis also suggested that DSS-induced UC mice had colonic injuries, such as a decrease in goblet cells, morphological changes in crypts, and inflammatory cells that diffused tissue infiltration, resulting in increased histopathological scores compared to those of the controls (Figures 3E, F). However, as shown in Figures 3E, F, the administration of 5-ASA or SJZD significantly improved colonic injury. Moreover, the ameliorating effect of SJZD on colonic injury appeared in a significant dose-dependent manner. The data confirmed that SJZD could ameliorate colonic injury.

### 3.4 SJZD-regulated inflammatory factor secretion

**FIGURE 4:**
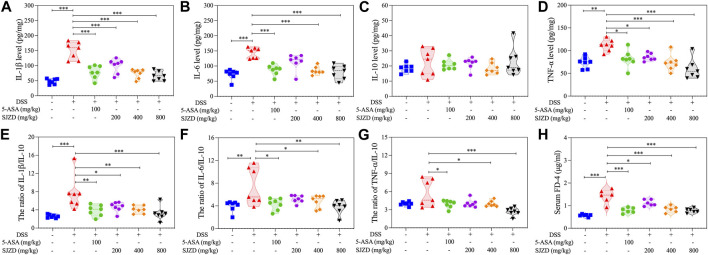
Regulation of SJZD on cytokines and intestinal permeability. **(A–D)** IL-1β, IL-6, IL-10, and TNF-α levels. **(E–G)** Ratio of IL-1β/IL-10, IL-6/IL-10, and TNF-α/IL-10. **(H)** FD-4 level in serum. The data were denoted in the form of mean ± SD (n = 6). *p < 0.05, **p < 0.01, and ***p < 0.001 relative to the DSS group.

The level of inflammatory factors has a significant impact on the progression of UC. In Figures 4A–D, the levels of pro-inflammatory factors (IL-6, TNF-α, and IL-1β) in the DSS group were observably increased compared with the controls, while the anti-inflammatory factor (IL-10) did not change significantly. In contrast, 5-ASA or SJZD intervention remarkably reduced tissue infiltration of these pro-inflammatory cytokines but had only little effect on the amount of anti-inflammatory cytokine expression (the change in anti-inflammatory factors was not obvious because the mice were in a state of stress.). To explore whether SJZD ameliorated UC by regulating colonic mucosal inflammation, we deeply analyzed the pro-inflammatory to anti-inflammatory cytokine ratio. In Figures 4E–G, increased secretion of pro-inflammatory cytokines after DSS intervention led to an inflammatory imbalance. However, IL-1β/IL-10, TNF-α/IL-10, and IL-6/IL-10 in colon tissue were remarkably reduced after treatment with 5-ASA or SJZD. These beneficial effects of SJZD also exhibited a clear dose-dependent pattern. The above results demonstrated that SJZD could regulate the colonic inflammatory response and attenuate inflammatory factor secretion.

### 3.5 SJZD enhanced the intestinal epithelial barrier function

**FIGURE 5:**
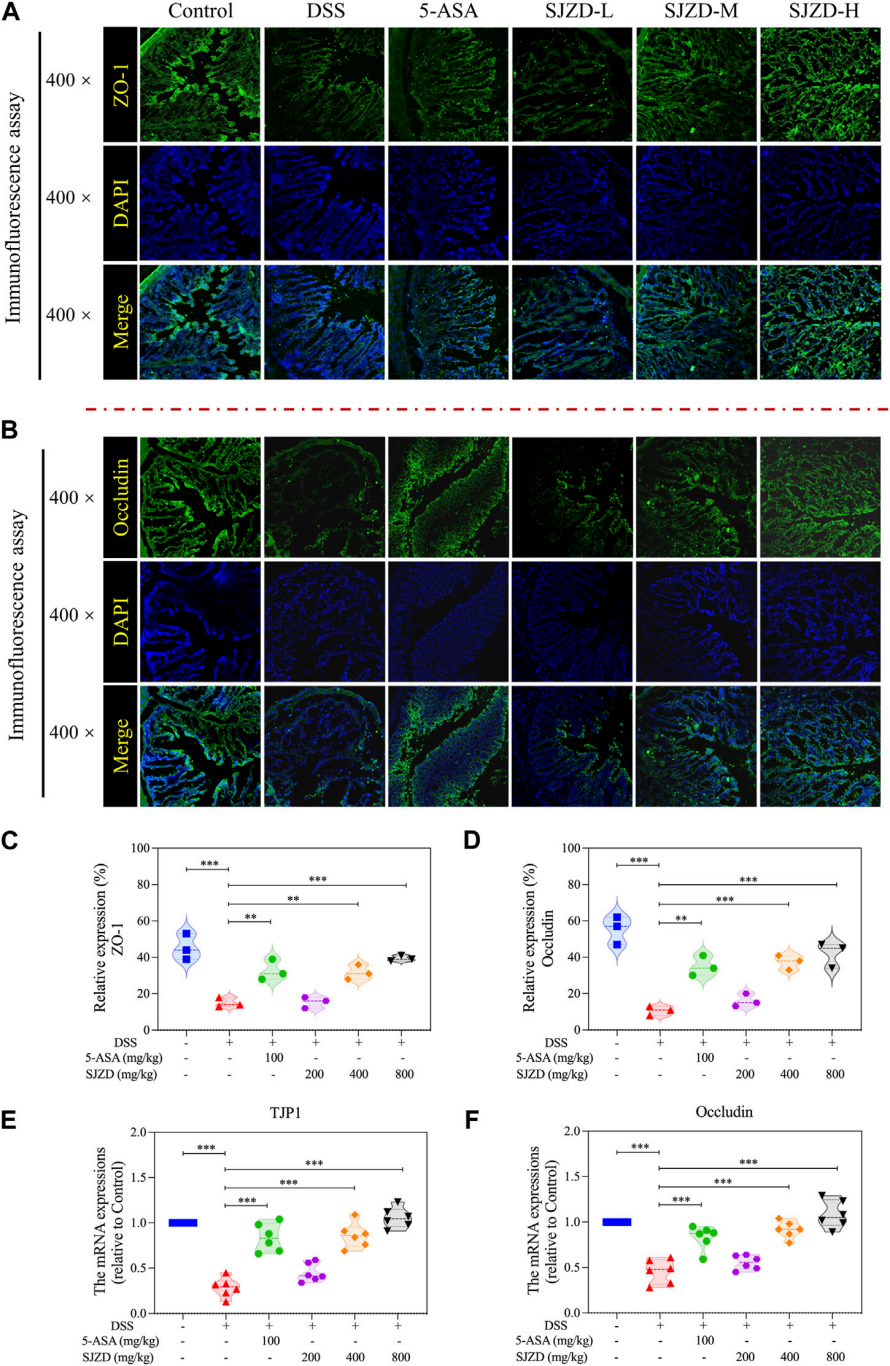
Expression of intestinal compact junction protein. **(A)** Representative immunofluorescence pictures of ZO-1 expression. **(B)** Representative immunofluorescence pictures of occludin expression. **(C)** ZO-1 expression level statistics. **(D)** Occludin expression level statistics. **(E)** mRNA expressions of TJP1 (relative to control). **(F)** mRNA expressions of occludin (relative to control). The data were denoted as the mean ± SD (n = 3). *p < 0.05, **p < 0.01, and ***p < 0.001 compared to the DSS group.

Increased intestine epithelial permeability is a marker of gut barrier dysfunction (Schoultz and Keita, 2020). Thus, serum FITC-Dextran (MW: 4,000) was detected to uncover whether SJZD could adjust intestinal barrier function by improving intestinal epithelial permeability. As shown in Figure 4H, there is an apparent increase in serum FITC-dextran in DSS-treated mice after 4 h of FITC-dextran intragastric administration, implying that DSS treatment could result in colonic barrier damage. Interestingly, SJZD (200, 400, and 800 mg/kg/d) groups in a dose-dependent manner attenuated serum FITC-dextran levels (Figure 4H). Occludin and ZO-1 both belong to tight junction proteins (Fang et al., 2021) and serve as mechanical barrier molecules that maintain intestinal mucosa integrity, thereby safeguarding intestinal homeostasis. The changes in the expression of ZO-1 and occlusion could directly mirror the intestinal barrier function. An immunofluorescence assay was applied to test occludin and ZO-1 in colon tissue. In Figures 5A–D, occludin and ZO-1 in the DSS group were inferior to those in the control group. In contrast, the expression of occludin and ZO-1 in the SJZD (200, 400, and 800 mg/kg/d) treated groups increased in a dose-dependent manner. Consistent with the expression of serum FITC-dextran, SJZD could enhance the intestinal barrier function to protect the tissue against UC. In addition, to further illustrate the effect of SJZD on the intestinal epithelial barrier, we performed a real-time PCR assay. As shown in Figures 5E, F, the mRNA expressions of occludin and TJP1 in the SJZD groups also increased in a dose-dependent manner. These results suggested that SJZD could enhance tight junction proteins and reduce colon permeability to induce the improvement of intestinal barrier function.

### 3.6 SJZD restored intestinal dysbacteriosis in the UC mouse model

**FIGURE 6:**
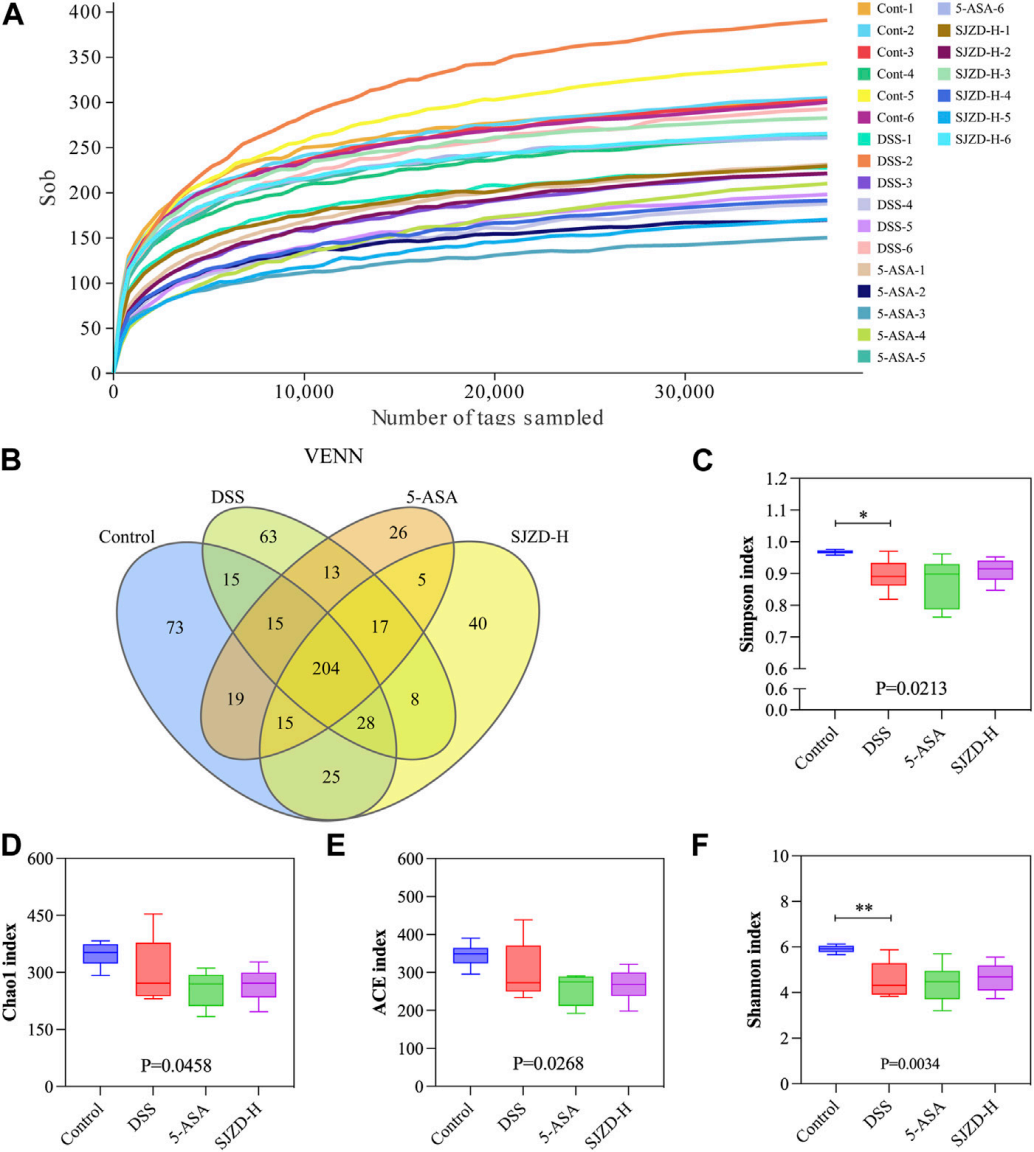
SJZD effect on gut microbiota diversity. **(A)** Dilution curves. **(B)** Venn diagram (n = 6). **(C–F)** Simpson index, Chao l index, ACE index, and Shannon index analysis.

Research has revealed that enteric microorganism disorders are related to decreased gut barrier function and the pathogenesis of UC (Guo et al., 2020; Wang et al., 2023). Hence, 16S rRNA sequencing was applied to draw the gut microbiota profile in UC mice and to investigate the interventional role of SJZD on it. The dilution curves are displayed in Figure 6A. Each curve in the dilution curve verges to a plateau, which indicates that the sequencing data are sufficient, and adding data only yields thimblefuls of new OTUs. On this basis, 566 OTUs were clustered from four groups of mouse fecal samples (control, DSS, 5-ASA, and SJZD-H groups), and then a VENN map was drawn using the OTU abundance table. As shown in Figure 6B, there were 204 common OTUs in the four groups. Our results indicate that relative to the control group, the diversity and abundance of enteric microorganisms in mice treated with DSS decreased (Figures 6C–F). Nevertheless, the SJZD intervention ameliorated the decrease in gut microbiota diversity (Figures 6C–F).

**FIGURE 7:**
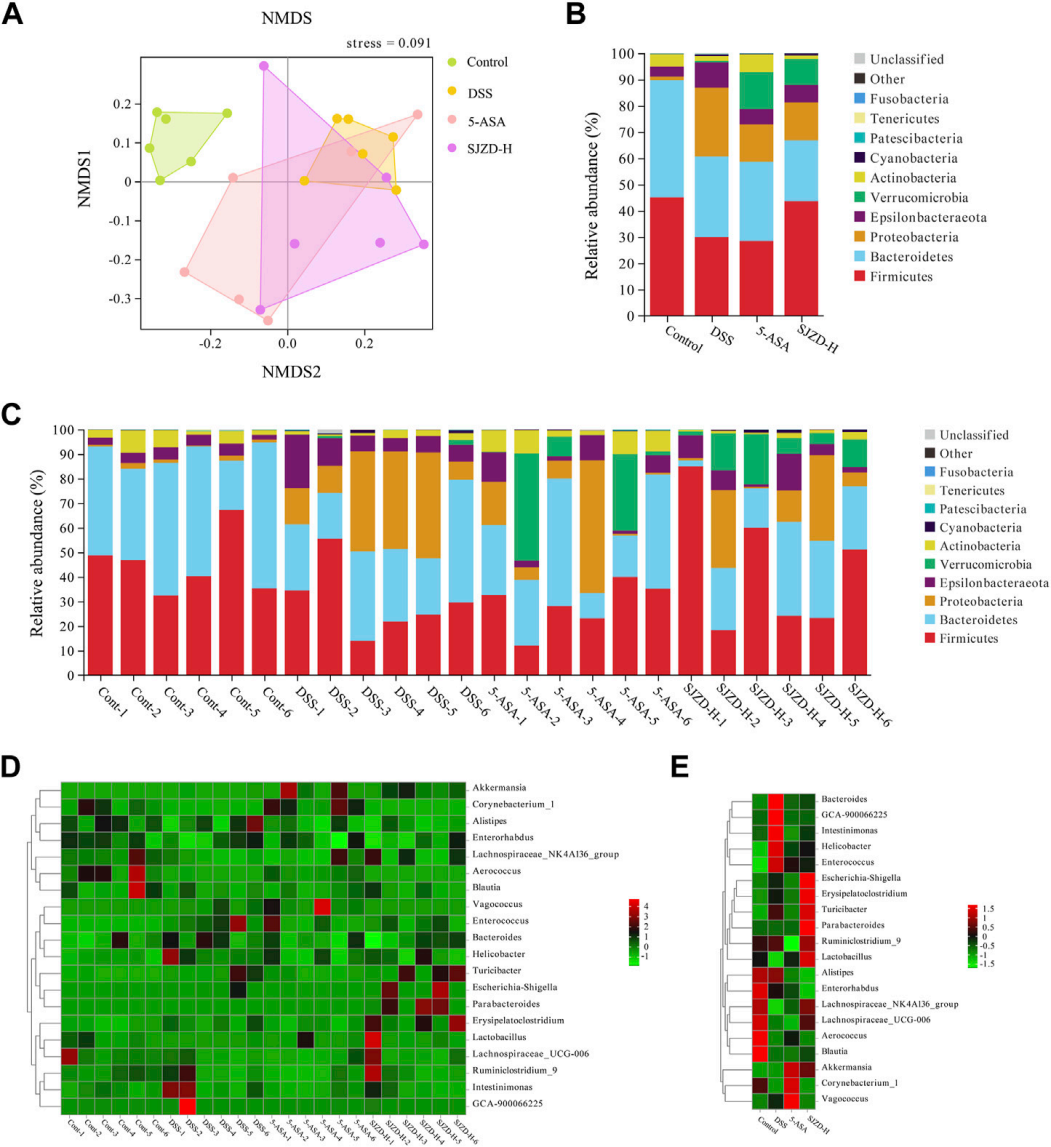
Effect of SJZD on gut microbiota diversity. **(A)** Non-metric multidimensional scaling analysis. **(B)** Top 10 bacteria at the phylum level between groups (n = 6). **(C)** Top 10 bacteria at the phylum level among samples (n = 6). **(D)** Top 10 bacteria at the genus level among samples (n = 6). **(E)** Top 10 bacteria at the genus level between groups (n = 6).

NMDS was used to compare the microbial differences between the groups. The results indicated a marked separation between the DSS and control groups, confirming a significant difference in the overall composition of the enteric microorganisms between the control and DSS groups (stress = 0.091 < 0.2; NMDS analysis results were reliable) (Figure 7A). Although there is a certain degree of crossover between the 5-ASA, SJZD, and DSS groups, some OTUs in the 5-ASA and SJZD groups are outside the range the area of the DSS group. Then, the microbial composition in UC mice was evaluated at both the phylum and genus levels. Figures 7B,C show the top 10 gut microbes at the phylum level. In comparison to the control group, the proportion of Bacteroidetes, Firmicutes, and Actinobacteria was reduced, while the Proteobacteria and Epsilonbacteraeota were increased in the DSS group. The 5-ASA or SJZD groups increased the abundance of Verrucomicrobia and Actinobacteria and decreased the abundance of Proteobacteria and Epsilonbacteraeota with respect to the DSS group. Interestingly, Verrucomicrobia was evidently enriched in the 5-ASA and SJZD groups compared to the control and DSS (Figures 7B,C). Meanwhile, the top 20 bacterial groups at the genus level are exhibited in Figures 7D,F. In comparison to the control group, the DSS group increased *Bacteroides*, *Helicobacter pylori*, and *Enterococcus* while reducing Leptospiraceae_NK4A136_ Group, Enterobacteriaceae, and Leptospiridae_ UCG-006. Furthermore, *Bacteroides* and *Helicobacter* in the SJZD group were much less than those in the DSS group. Erysipelotrichia, Turicibacter, Parabacteroides, *Lactobacillus*, Lachnospiraceae_NK4A136_group, Akkermansia, and Lachnospiraceae_UCG-006 were more abundant in the SJZD groups compared to the DSS group (Figures 7D–F). The data implied that SJZD could remodel gut microbiota homeostasis to a certain extent.

**FIGURE 8:**
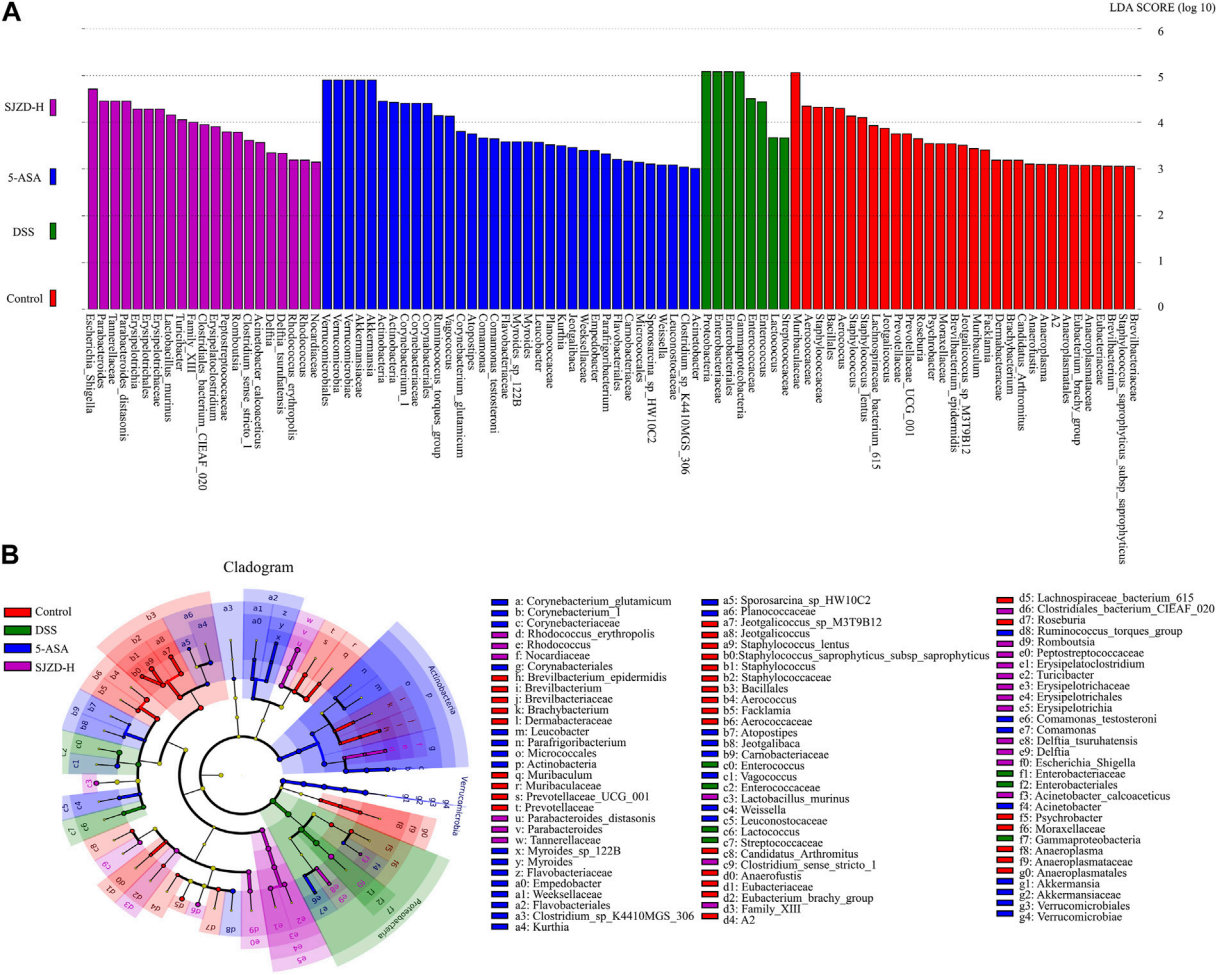
SJZD effect on the differential markers of the microbiota. **(A)** LDA scores histogram (LDA>2.0) (n = 6). **(B)** LEfSe analysis cladogram (n = 6).

Linear discriminant analysis effect size analysis (LEfSe) obtained the evolutionary branch diagram and LDA value distribution histograms shown in Figures 8A,B (LDA >2, *p* < 0.05). Enterobacteriaceae, Enterobacteriales, Gammaproteobacteria, Enterococcaceae, and *Enterococcus* were enriched in the DSS group. It can be used as a biomarker for the DSS group. Verrucomicrobiales, Verrucomicrobiae, Verrucomicrobia Akkermansiaceae, Akkermansia, Actinobacteria, Corynebacterium_1, Corynebacteriaceae, and Corynebacteriales were enriched in the 5-ASA group and could be used as biomarkers in the 5-ASA group. At the same time, *Escherichia*_*Shigella*, Parabacteroides, Tannerellaceae, Parabacteroides_distasonis, Erysipelotrichia, Erysipelotrichales, Erysipelotrichaceae, and *Lactobacillus*_murinus were enriched in the SJZD group and could be used as biomarkers of SJZD. Gut microbiota, a new biomarker, is crucial in maintaining intestinal homeostasis (Chen et al., 2020). Bacteroidetes (Wang et al., 2021a), *Lactobacillus* (Pang et al., 2021), Muribaculaceae (Hiraishi et al., 2022), Verrucomicrobia (Kim et al., 2021), Akkermansia (Si et al., 2022), and Erysipelotrichaceae (Mao et al., 2021) can regulate pro-inflammatory factor levels, short-chain fatty acids (SCFAs), and butyrate production to maintain the gut barrier. Thus, the increase of these probiotic bacteria is a pivotal sign reflected in UC recovery after SJZD intervention. Meanwhile, the lack of probiotics or the presence of pernicious bacteria such as Enterobacteriaceae (Lupp et al., 2007), Proteobacteria (Wang et al., 2021b), *Helicobacter* (Salvatori et al., 2023), and *Enterococcus* (Fiore et al., 2019) may elevate the risk of amplifying inflammation and intestinal epithelial injury. Taken together, SJZD could modulate or reverse the gut microbiota composition.

### 3.7 Effect of SJZD on gut microbiota functions

**FIGURE 9:**
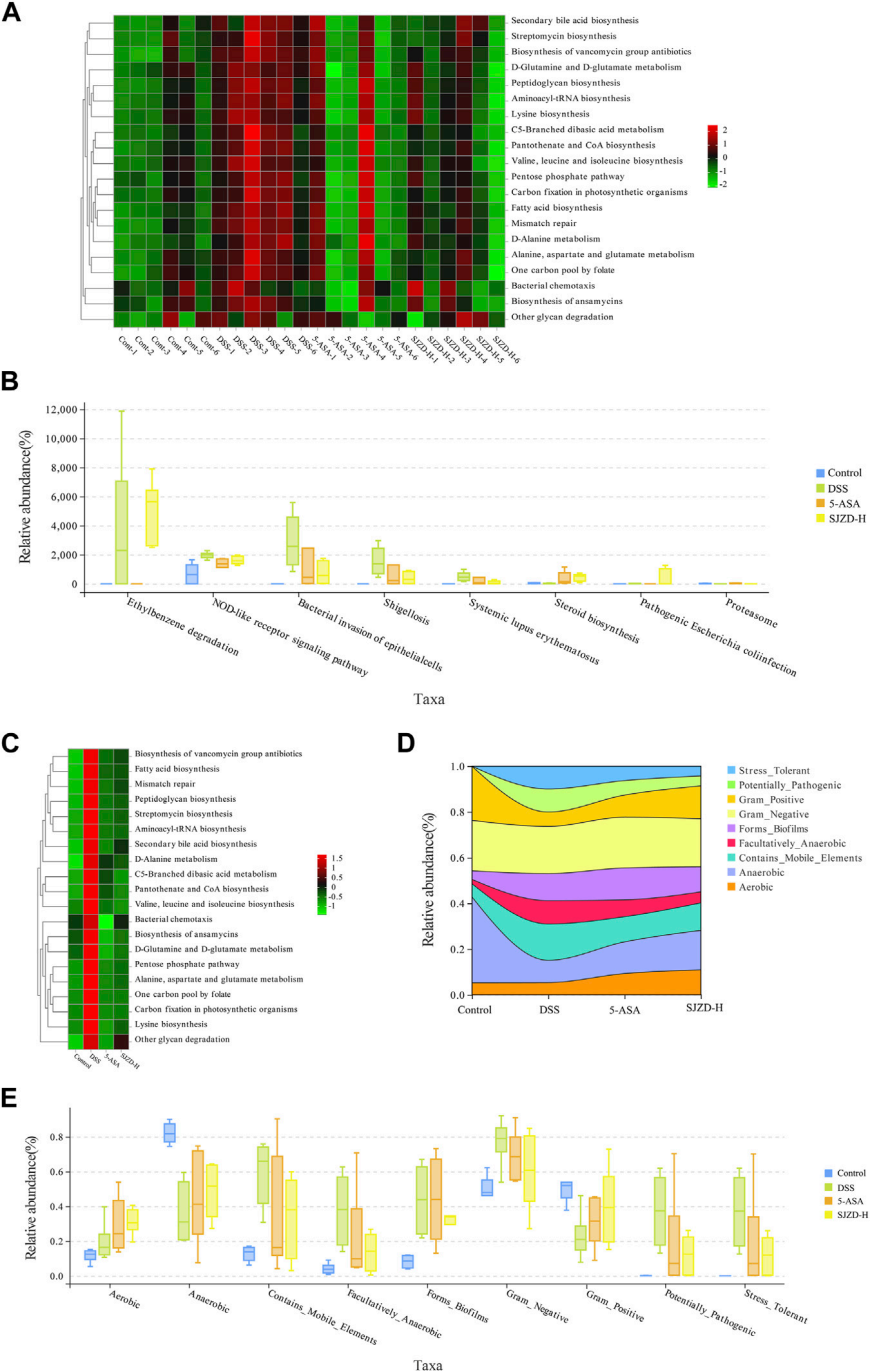
SJZD effect on microbiota function and phenotype in UC mice gut. **(A)** Abundance heatmap of microbiota function among samples (n = 6). **(B)** Kruskal–Wallis analysis for the microbiota function (n = 6). **(C)** Abundance heatmap of the microbiota function between groups (n = 6). **(D)** Stream analysis plot of phenotypic abundance. **(E)** Wilcoxon analysis of the microbiota phenotype (n = 6).

Enteric microorganisms play an essential role in an organism’s physiology and disease development and can also regulate biological functions *in vivo* (Wu et al., 2021). In this study, PICRUSt2 analysis further predicts the impact of SJZD on microbiota functions (Figures 9A,C). Metabolism, a functional category, was mainly affected and increased in relative abundance after DSS intervention, thereby participating in the pathogenesis and progression of UC. The abundance of metabolic markers, such as secondary bile acid biosynthesis, streptomycin biosynthesis, peptidoglycan biosynthesis, D-glutamine, and D-glutamate metabolism, and the biosynthesis of vancomycin-group antibiotics, was notably increased in the DSS group compared to the controls. However, SJZD intervention successfully suppressed the abundance of these metabolic markers, but the results were not significantly different (Figure 9B).

The impact of SJZD on the microbiota’s phenotypic function was explored based on BugBase analysis. As shown in Figures 9D,F, Stress_Tolerant, Contains_Mobile_Elements, Forms_Biofilms, Facultatively_Anaerobic, Contains_Mobile_Elements, and Potentially_Pathogenic had a higher abundance ratio in the DSS group compared to the controls. The abundance of Gram_Negative and Aerobic did not change much, while that of Gram_Positive and Anaerobic decreased. Interestingly, in comparison with the DSS group, the abundance ratio of Stress_Tolerant, Potentially_Pathogenic, Facultatively_Anaerobic, and Contains_Mobile_Elements was reduced after 5-ASA and SJZD treatment. Meanwhile, the relative abundance levels of Gram_Positive, Forms_Biofilms, Anaerobic, and Aerobic were enriched. These results indicated that SJZD could modulate the microbiota phenotype and functions in the intestine of UC mice.

### 3.8 Analysis of data distribution characteristics

**FIGURE 10:**
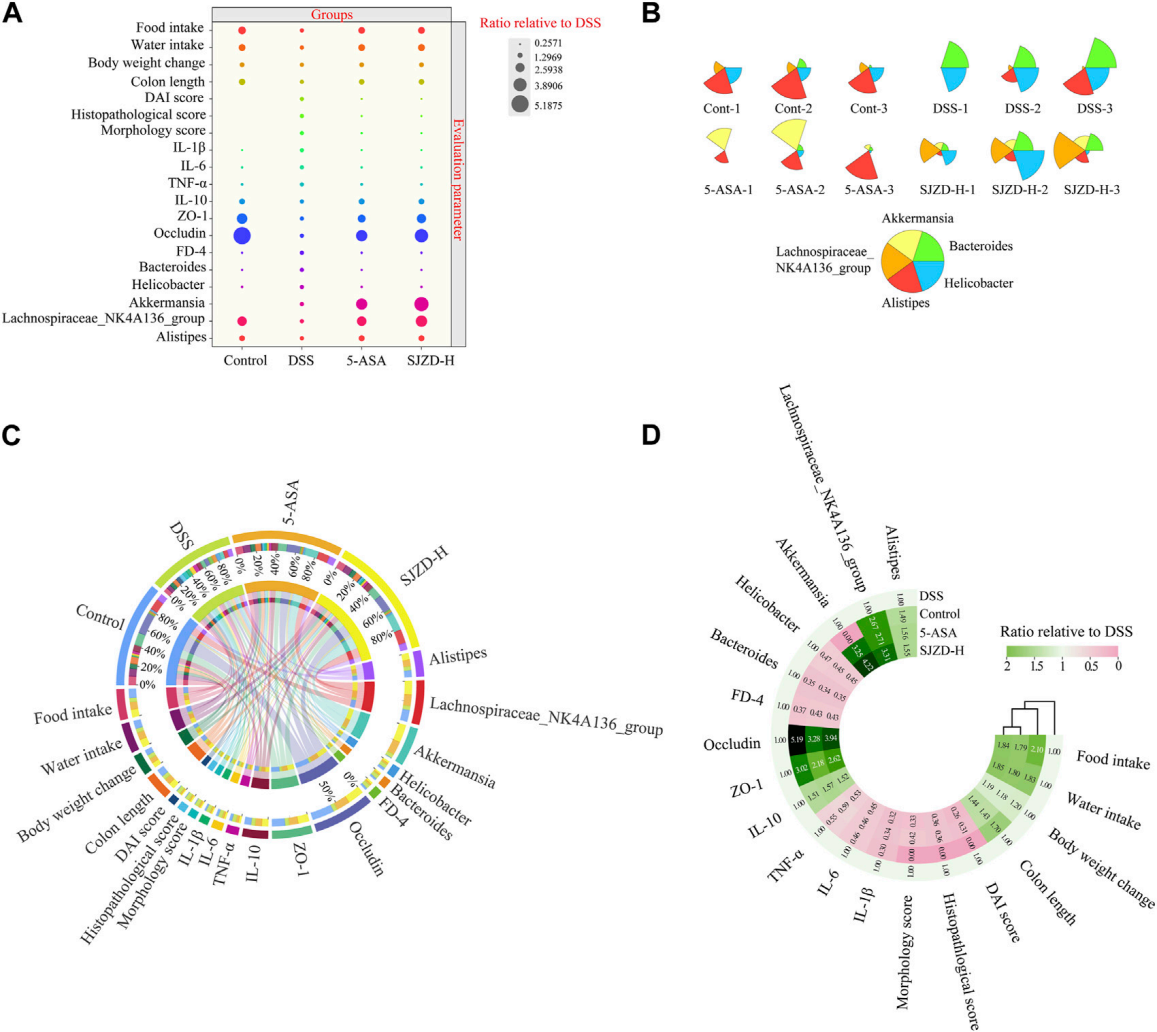
Data distribution, cluster analysis, and correlation analysis. **(A)** Bubble plot of the normalized distribution relative to DSS. **(B)** Star map of the top five relative abundances at the genus level among samples. **(C)** Circos diagram of the normalized distribution relative to DSS. **(D)** Cluster heatmaps of the normalized distribution relative to DSS.

Based on the clinical symptom parameters, inflammatory factors, pathological scores, intestinal barrier function, intestinal epithelial permeability indicators, and gut microbiota, the data of each group were standardized relative to the DSS group, and then statistical and cluster analyses were performed to investigate the regulation of data variation. As illustrated in Figures 10A,C, there were clear differences in the distribution of data on intestinal barrier function, intestinal epithelial permeability, and gut microbiota between different interventions. Meanwhile, the cluster analysis in Figure 10D showed that the indicators of each group showed an obvious clustering phenomenon. Broadly speaking, the data in the different treatment groups were classified into two clusters. Therein, DSS belongs to one cluster, while control, 5-ASA, and SJZD belong to the other cluster. The above results suggested that SJZD could reverse DSS-induced UC.

### 3.9 Correlation analysis between epithelial barrier, inflammation, and gut microbiota

**FIGURE 11:**
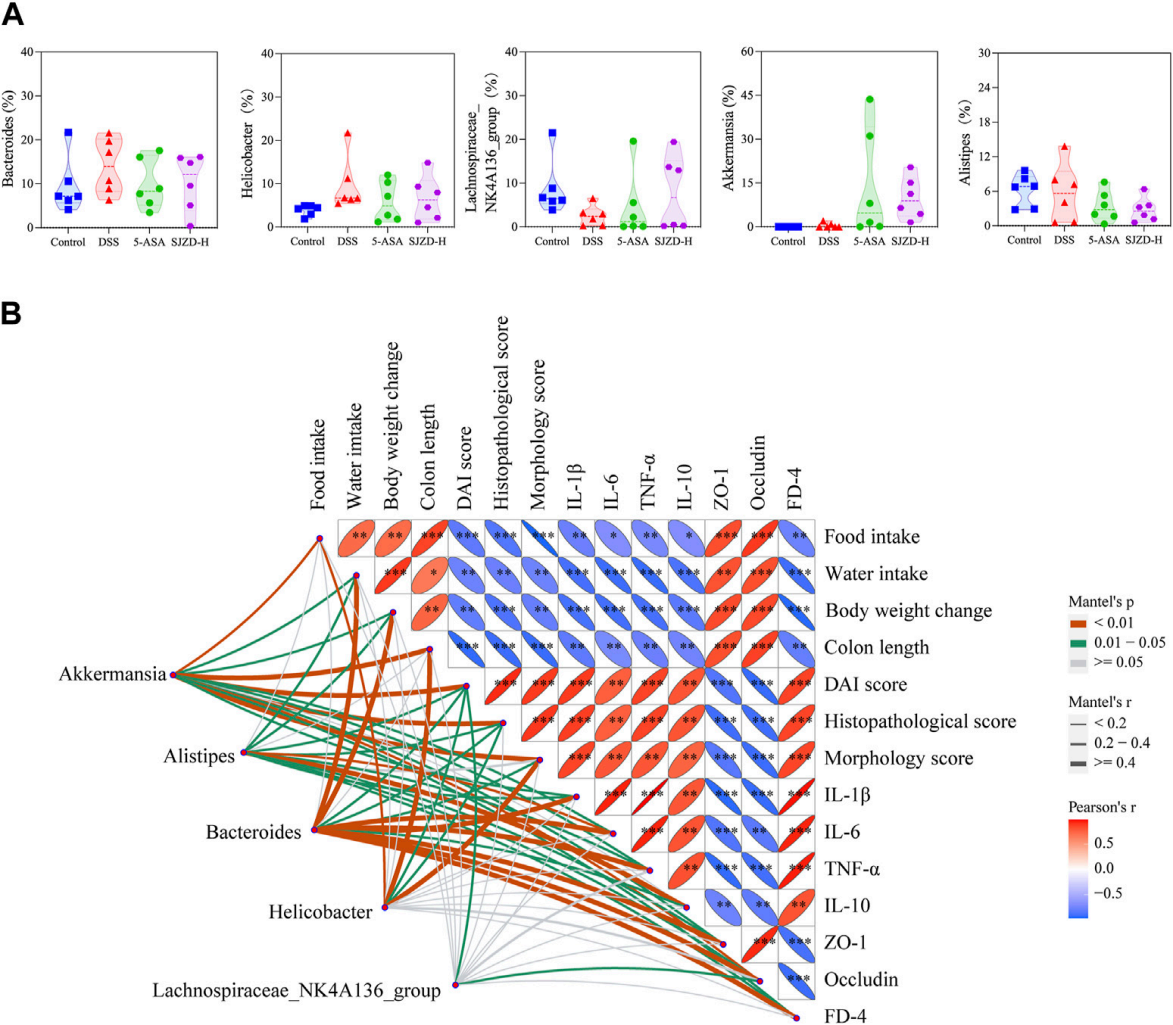
Pearson correlation analysis and differential genus-level microbiota. **(A)** Relative abundance (%) of *Helicobacter*, *Bacteroides*, Lachnospiraceae_NK4A136_group, Akkermansia, and Alistipes. **(B)** Correlations among differential genus-level microbiota, UC clinical parameters, inflammatory cytokines, gut mucosal barrier, and permeability indicators. The data were denoted as the mean ± SD (n = 5) pattern. *p < 0.05, **p < 0.01, and ***p < 0.001 relative to the DSS group.

**FIGURE 12:**
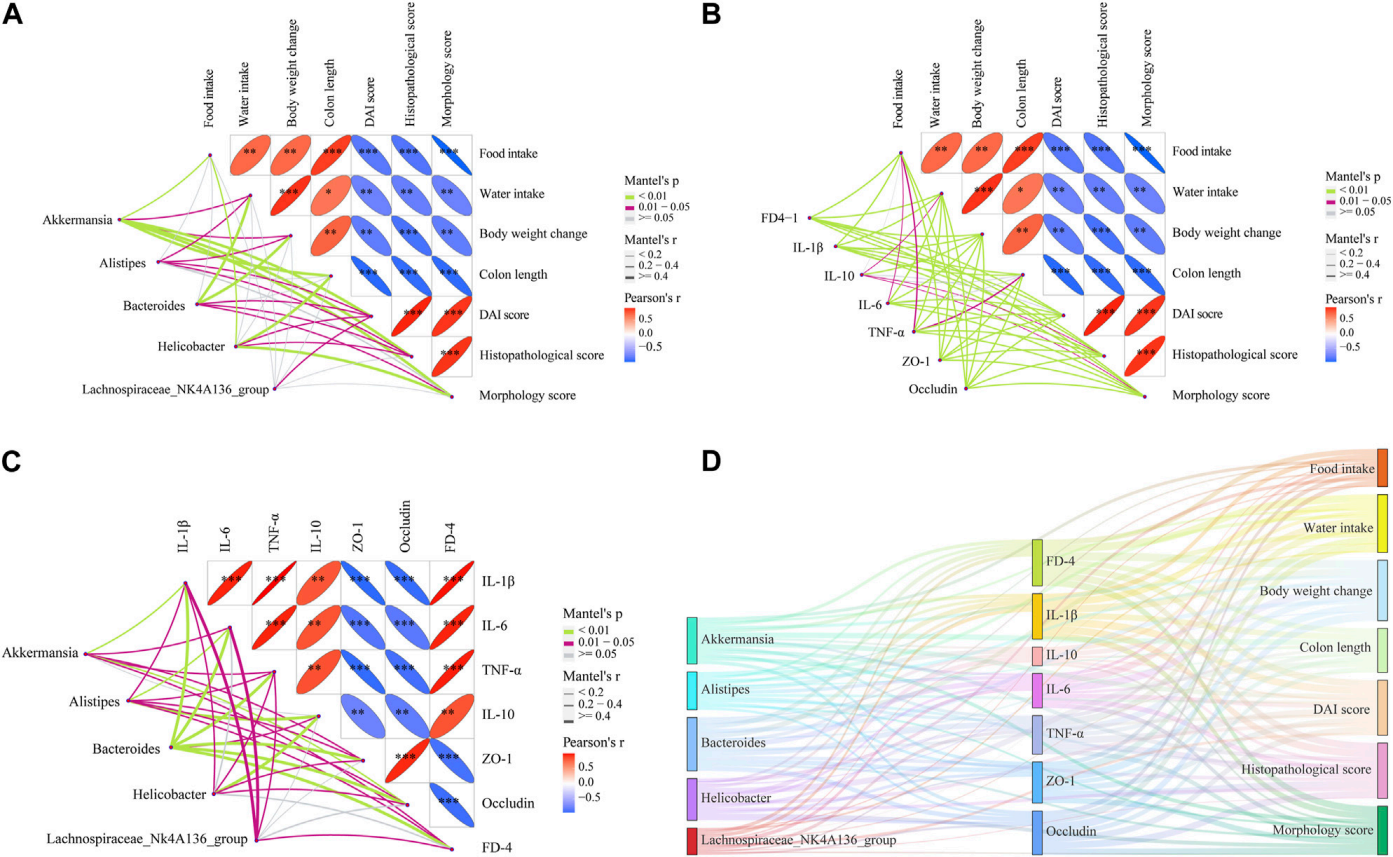
Correlations between multiple data points. **(A)** Correlations between significant flora and clinical parameters of UC. **(B)** Correlation analysis among UC clinical parameters, inflammation factors, gut barrier, and permeability indicators. **(C)** Correlations between significant flora, inflammation, gut barrier, and permeability indicators. **(D)** Sankey diagram of Pearson coefficients between significant flora, UC clinical parameters, inflammation, gut barrier, and permeability with each other. *p < 0.05, **p < 0.01, and ***p < 0.001 relative to the DSS group.

According to the LEfSe analysis, among bacteria with different characteristics (LAD >2), the genus level occupied the largest proportion, which implied that the genus level played a crucial role in this research. On this basis, the top five genus-level bacteria with the highest relative abundance were selected for analysis (Figures 10B, 11A). Compared to the control group, the abundance of *Bacteroides* and *Helicobacter* increased, while that of Lachnospiraceae_NK4A136_group and Alistipes decreased in the DSS group. Interestingly, there was little change in Akkermansia between the control and DSS groups. Compared with the DSS group, the SJZD group increased the abundance of Akkermansia and Lachnospiraceae_NK4A136_group while decreasing that of *Bacteroides* and *Helicobacter*. In addition, the correlation between genus-level key microbiota and clinical parameters (such as UC characteristics, inflammatory factors, intestinal barrier, and permeability) was investigated to determine the latent effects of gut microbiota in UC (Figure 11Β). The results found that *Bacteroides*, Lachnospiraceae_NK4A136_group, Akkermansia, Alistipes, and *Helicobacter* were strongly correlated with clinical characteristics, inflammatory factors, intestinal barrier proteins, and intestinal epithelial permeability of UC. These outcomes indicated that those microorganisms play an essential role in UC. However, whether there is a correlation between the different indicators is unknown. Hence, we further investigated the correlation between the significantly enriched microbes, inflammatory cytokines and gut barrier proteins, and clinical parameters based on Pearson correlation analysis, respectively. As shown in Figures 12A,B, the genus-level significant microbiota, inflammatory factors, and intestinal epithelial barrier markers were both positively correlated with clinical features (food ingestion, water ingestion, body weight variation, and colon length). In contrast, these indices were negatively correlated with colonic injury parameters (DAI score, histopathology score, and morphology score). To ascertain the underlying association between gut microbiota, gut barrier proteins, and inflammatory cytokines, Pearson correlation analysis was performed on these indicators (Figure 12C). The results displayed that the gut microbiota was remarkably correlated with inflammatory cytokines and gut barrier proteins. Similarly, the Sankey plot showed that there was a prominent correlation between clinical parameters, gut microbiota, inflammatory factors, and intestinal epithelial barrier markers (Figure 12D). Therefore, the results presented by Pearson’s analysis tentatively suggested that SJZD alleviated UC by alleviating gut microbiota-mediated inflammation and intestinal epithelial barrier damage.

## 4 Discussion

Increasing evidence suggests that the pathogenesis of UC is connected with hereditary factors, intestinal barrier injury, inflammatory disequilibrium, and microbial dysregulation. Based on the reported regulatory effect of gut microbiota by SJZD in disease prophylaxis and treatment, this paper proposed a reasonable hypothesis that SJZD alleviates UC via improving gut microbiota-mediated colorectal barrier dysfunction and inflammatory disequilibrium. Our research findings found that SJZD could reduce inflammation, restore colorectal barrier function, and improve the enteric permeability of UC mice by restoring intestinal microbial balance. These data proved the feasibility of this hypothesis with indisputable facts.

SJZD has been used in the clinical practice of gastrointestinal diseases for more than 1,000 years (Huang et al., 2017). In the preliminary preparation work, the research group conducted a systematic study on the extraction, concentration, and drying of SJZD and established a standardized preparation process. To ensure the stability of SJZD, the present study investigated its chemical metabolites using UHPLC-QTOF/MS. Thus, the controllability and reproducibility of this study were ensured. Based on the TCM theory, the primary pathogenesis of UC involves spleen–stomach weakness and disorders of transport and digestion, which will dissipate heat and change fire as time passes. It follows that spleen-qi insufficiency is the fundamental reason for the pathogenesis and pathogenic processes of UC. Therefore, SJZD, as a traditional prescription for invigorating the body and strengthening the spleen, should be the first choice for the therapeutic regimen of UC (Tian et al., 2021). Our findings confirmed that SJZD can indeed effectively prevent and treat UC.

Previous studies have shown that the DSS-induced UC mouse model and UC patients have extremely similar clinical manifestations (weight loss, bloody stool, and loose stool) and histological changes (inflammatory factor infiltration, intestinal epithelial barrier damage, and increased intestinal epithelial permeability) (Guo et al., 2022). Therefore, the mouse model of DSS-induced UC is the most widely applicable and easily described IBD model (Dong et al., 2022b). Based on this, the impact of SJZD on UC mice could be evaluated by key indicators such as clinical parameters and histopathological changes. The research findings showed that the DSS group showed clinical characteristics such as bloody stool, loose stool, diarrhea, and weight loss, which could be regarded as successful modeling. SJZD could reduce these clinical characteristics in a dose-dependent manner. DSS could inhibit enterocyte proliferation and damage intestinal immunologic function to cause epithelial barrier dysfunction, colon length shortening, and colon inflammation, which eventually leads to UC (Yu et al., 2016). Interestingly, SJZD and 5-ASA have similar therapeutic effects in inhibiting colonic atrophy, reducing intestinal mucosal edema, and preventing ulceration in mice. All those results suggested that SJZD could be a vital drug to alleviate UC. However, the UC mouse model used in our study was an acute UC model caused by DSS, which did not reflect the dynamic process of disease progression compared to the chronic UC model. Hence, the impact of SJZD on DSS-induced chronic UC remains to be further studied.

UC is characterized by diffuse injury to the colonic mucosa and an imbalance of inflammation (Dong et al., 2022a). The integrity of the intestinal epithelial barrier is influenced by the inflammatory response. For example, inflammatory factors can aggravate tissue infiltration of neutrophils and macrophages. The imbalance in the inflammatory response contributes to the destruction of the intestinal barrier, edema of the intestinal cavity, erosion of the large intestinal mucosa, and finally the formation of ulcers (Li et al., 2020). Therefore, the inflammatory response is a key link in UC pathogenesis. Several studies have found that the presence of pro-inflammatory cytokines and an imbalance in anti-inflammatory cytokines hinder the elimination of inflammation (Wang et al., 2021a). Our research uncovered that 5-ASA or SJZD dramatically reduced the infiltration of inflammatory factors into mucosal or submucosal tissues. In addition, SJZD in a dose-dependent manner decreased pro-inflammatory cytokines. Although 5-ASA or SJZD did not noticeably affect IL-10, it obviously decreased IL-6/IL-10, IL-1β/IL-10, and TNF-α/IL-10. Taken together, these data confirmed that SJZD could attenuate UC colonic injury by attenuating inflammation, and the effect of ameliorating colonic injury was dose-related.

The intestinal barrier is critical to protecting the host from invasion by exogenous pathogens. The destruction of this barrier may result in an increase in intestinal epithelial permeability and intestinal leakage and promote the release of pathogenic bacteria and toxins from the intestine into the blood (Guo et al., 2022). Tight junctions are the structural basis of the mechanical barrier. Membrane-spanning protein (occludin) and cytosolic protein (ZO-1) are involved in the construction of tight junctions (Fang et al., 2021). The tight junction is a significant component in the maintenance of the homeostatic balance of the colorectum, which is able to resist pernicious substances and microorganisms. The DSS-induced UC model is characterized by intestinal epithelial cell injury and intestinal barrier dysfunction (Rui et al., 2021). The present study showed that ZO-1 and occludin proteins were evidently upregulated after SJZD intervention. The upregulation of tight junction protein expression, a major determinant of intestinal barrier function, offers another description of the SJZD effect on anti-UC. Thus, SJZD has a favorable impact on intestinal barrier reconstruction in addition to the anti-inflammatory response.

The enteric microorganism constitutes the intestinal microbial barrier. Increasing evidence has shown that gut microbiota plays a pivotal role in the pathogenesis and progression of UC (Guo et al., 2020; Wang et al., 2023). When the homeostasis between pathogenic and probiotic bacteria is destroyed, the pathogen-induced inflammatory response damages the tight junctions between enterocytes (Lu et al., 2022). Meanwhile, the intact epithelial barrier function hinges on the mutual effects among enteric microbes (Guo et al., 2022). In short, enteric dysbacteriosis results in an increase in enteral mucosal permeability, the release of inflammatory cytokines, and the induction of gut barrier dysfunction, which then promotes the pathogenesis and progression of UC. The imbalance of the gut microbiota is the initial link and persistent factor for UC (Tian et al., 2021). Therefore, re-establishing microbial homeostasis has been identified as one of the key therapies for UC. It has been reported that a variety of drugs can alleviate UC by remodeling the gut microbiota (Feng et al., 2022; Wang et al., 2023). Based on this, we explored whether SJZD has an advantage in reconstructing the microbiota in the UC mouse model. According to the reports, the increased relative abundance of Proteobacteria (Rizzatti et al., 2017), *Helicobacter* (Salvatori et al., 2023), Enterobacteriaceae (Baldelli et al., 2021), and *Enterococcus* (Fiore et al., 2019) is strongly associated with the massive activation of inflammatory responses.

Proteobacteria, Epsilonbacteraeota, *Bacteroides*, *Helicobacter*, and *Enterococcus* were enriched in the DSS group, suggesting an imbalance of the gut microbiota in mice after DSS intervention. Bacteroidota, Deferri-bacterota, Proteobacteria, Actinobacteria, Firmicutes, and Desulfobacterota were the six center phyla of the gut microbiota, of which Bacteroidetes and Firmicutes accounted for over 95% of all (Wang et al., 2022). The literature has demonstrated that Firmicutes/Bacteroidetes are positively related to intestinal health (An et al., 2023). The present study shows that SJZD could markedly increase the Firmicutes/Bacteroidetes ratio compared to DSS, which implies that SJZD could regulate the transformation of enteric microorganisms into normal gut microbiota and promote gut health. Butyrate, the dominant energy source for enterocytes, can stimulate enterocytes to produce mucus, rearrange tight junction proteins, and improve intestinal barrier function (Silva et al., 2018). In this study, SJZD enriched several butyrate-producing florae, such as Lachnospiraceae_NK4A136_group, Muribaculaceae (Dong et al., 2022b), and Lachnospiraceae_UCG-006. However, some pernicious bacteria with higher abundances were also found in the SJZD group, such as Erysipelotrichia. In addition, Akkermansia, a bacterium of the phylum Verrucomicrobia that uses mucin as the main energy source, is considered to be a promising probiotic candidate for reducing inflammation, maintaining intestinal integrity, and ameliorating host metabolism and immunity (Si et al., 2022). We found that Verrucomicrobia was enriched after treatment with 5-ASA and SJZD compared with DSS treatment. Our research suggested that SJZD could reverse the imbalance of gut microbiota by enriching probiotics or lessening pathogenic bacteria, thereby treating UC.

PICRUSt and BugBase were applicable to forecasting the intestinal function and phenotype of the microbiota (Röttjers and Faust, 2018). Although these two methods have limitations in some aspects, PICRUSt gains the KEGG homology/pathway abundance tables of samples (Douglas et al., 2020), and BugBase offers 16S-based annotation and community genomics datasets. For this paper, DSS mainly affected metabolic function categories, and most of these effects were eliminated after SJZD intervention. Concurrently, the phenotypes of the control, 5-ASA, DSS, and SJZD groups were significantly different. The relative abundance of Stress_Tolerant, Potentially_Pathogenic, Facultatively_Anaerobic, and Contains_Mobile_Elements was higher in the DSS group. After 5-ASA and SJZD treatment, these phenomena could be improved, and the relative abundance of Gram_Positive, Forms_Biofilms, Anaerobic, and Aerobic bacteria increased.

In order to understand the distribution of the study data, we conducted data statistics and clustering analysis to find the data distribution rule. The results confirmed that the distribution of indicators in different intervention groups was different and that there were obvious clustering phenomena. Among them, DSS belongs to one cluster, while the control, 5-ASA, and SJZD groups belong to another cluster. It is worth mentioning that in the control, 5-ASA, and SJZD clusters, the control group belongs to one category, while the 5-ASA and SJZD groups belong to the other, which indicates that SJZD has the same effect as 5-ASA in relieving UC. To elucidate the latent effect of the gut microbiota in UC, we performed a correlation analysis between clinical parameters of UC and genus-level important flora. The literature indicates that the correlation gets stronger as the coefficient approaches an absolute value of 1 (Schober et al., 2018). Our studies showed that *Bacteroides*, *Helicobacter*, Alistipes, Akkermansia, and Lachnospiraceae_NK4A136_group were strongly correlated with UC characteristics, inflammatory factors, and intestinal barrier protein, which suggests that SJZD could improve UC by regulating the gut microbiota. To further clarify the relation between significant microbiota, intestinal barrier proteins, and inflammatory factors, we performed Pearson correlation analysis on these indicators separately. The results implied a strong correlation among epithelial barrier proteins, inflammatory factors, and gut microbiota. Therefore, we can tentatively conclude that SJZD can alleviate UC by improving gut microbiota-mediated inflammation and intestinal barrier impairment.

Some limitations arising from limited time and space should be mentioned. For example, the extract of SJZD in the present research was a mixture of several metabolites. However, the most effective anti-UC plant metabolite and its absorption and metabolism *in vivo* are still unknown. Due to the short administration time of SJZD administration in this experiment, whether there is organ toxicity after long-term administration of SJZD has not been elucidated. Furthermore, the exact mechanism of the SJZD pathway for the treatment of UC should be further investigated in detail. Therefore, future studies could place emphasis on the isolation and metabolism of key metabolites of SJZD and explore the mechanism of SJZD-anti-UC in depth by gene silencing or gene knockout experiments. Moreover, we also hope, in the future, to use metagenomics sequencing techniques or fecal microbiota transplantation experiments to further determine the role of gut microbiota in the treatment of SJZD.

## 5 Conclusion

In conclusion, SJZD has a significantly curative effect on DSS-induced acute UC. The relieving effect of SJZD on UC was achieved by regulating the gut microbiota. The regulation of SJZD in the gut microbiota can inhibit inflammation, remodel the intestinal barrier, reduce intestinal epithelial permeability, and alleviate the clinical symptoms of UC. Therefore, our findings suggest that SJZD, as a potential medicine for UC, offers a novel curative approach for relieving UC symptoms in modern medicine.

## Data Availability

The datasets presented in this study can be found in online repositories. The names of the repository/repositories and accession number(s) can be found in the article/Supplementary Material.

## Abbreviations

5-ASA, 5-aminosalicylic acid; BPC, base peak chromatogram; DAI, disease activity index; DSS, dextran sodium sulfate; FD-4, FITC-dextran (MW: 4,000); FITC, fluorescein isothiocyanate; H&E, hematoxylin and eosin; IBD, inflammatory bowel disease IL-10, interleukin-10; IL-1β, interleukin-1β; IL-6, interleukin-6; LEfSe, linear discriminant analysis effect size; NMDS, non-metric multidimensional scaling analysis; SCFAs, short-chain fatty acids; SD, standard deviation; SJZD, Sijunzi decoction; TCM, traditional Chinese medicine; TJP1, tight junction protein 1; TNF-α, tumor necrosis factor-α; UC, ulcerative colitis.
